# Safety enhancement in adult body computed tomography scanning: comparison of iodixanol versus iohexol

**DOI:** 10.1186/s40064-016-1754-z

**Published:** 2016-02-24

**Authors:** Yong Xiao, Guofei Zeng, Xi Liu, Cong Peng, Changsu Lai, Peihua Zhou

**Affiliations:** Department of Radiological, Chongqing City Hospital of Traditional Chinese Medicine, 400021 Chongqing, China

**Keywords:** Iodixanol, Iohexol, Body computed tomography (CT), Adverse events

## Abstract

**Background:**

The aim of this study was to compare the safety and intra-individual contrast enhancement of low-osmolar monomeric iohexol and the iso-osmolar dimeric iodixanol in body computed tomography (CT) scanning.

**Methods:**

In this single center, double-blind, prospective study, a total of 2000 consecutive patients undergoing adult body CT scanning were prospectively enrolled, with 1000 patients assigned to iodixanol and 1000 patients assigned to iohexol. In both groups, the contrast medium was injected at the rate of 3.5 ml/s. Subjective assessment of image quality for each image was determined using a 3-grading scale by three reviewers. Patients were monitored with questioning and vital signs before injection, immediately after injection, and at 24 and 48 h. Extensive laboratory evaluation also was performed.

**Results:**

Laboratory results showed no significant difference across groups. There were also no detectable differences in image quality between the two contrast groups in this study. The total adverse reactions occurred in less than 1 % of individuals receiving iodixanol comparing to 2.5 % in iohexol group (P < 0.05). Among them, only 0.7 % patients injecting iodixanol suffered immediate events, comparing to 2 % patients in iohexol group. In all, 0.2 % patients with iodixanol and 0.5 % with iohexol had late allergic reaction. Further, No deaths occurred in any of the two groups.

**Conclusions:**

The iso-osmolar iodixanol provides image quality compared with that of iohexol, with lower incidence of adverse events.

## Background

The intravenous injection of contrast materials for medical imaging is usually a safe procedure and has been widely used throughout the world, over the last few decades (Casserbaum 1965). However, some individuals may undergo discomfort, experience allergy-like reactions or even adverse events (Becher et al. 2005). The hyper-osmolality is believed to be an important factor for systemic adverse reactions of contrast agents and responsible for local symptom from contrast media extravasations (Lee et al. 1996). More efficacious and safe contrast agents have been extensive developed to benefit the patients’ care in terms of their decreasing osmolality.

Iodixanol (Hengrui, Jiangsu, China) is a water-soluble, nonionic, dimeric, isosmolar radiographic material for intravascular injection (Spencer and Goa 1996). Preclinical researched have detected better toxicological pharmacological and physicochemical properties of iodixanol (Wilcox et al. 1987). A latter double blind, randomized, prospective, multi-center study has concluded that iohexol was more nephrotoxic among high risk groups, compared with the iso-osmolar, agent iodixanol (Doerfler et al. 1999). Numerous clinical trials also demonstrated comparable safety and efficacy in body computed tomography usage (Lee et al. 1996). Iohexol is another extensively used low-osmolar monomer non-ionic contrast medium, which is considered to safe, efficient and be well-tolerated for the head and body computed tomography (Almen 1983).

Therefore, we try to perform an intra-individual evaluation of the degrees of image quality and safety enhancement achieved by iohexol and iodixanol in body computed tomography (CT) scanning under the standardized conditions. Patients were assigned to contrast medium groups randomly.

## Materials and methods

### Patients

This was a randomized study conducted from April 2014 to 2015 at Chongqing City Chinese Medicine Hospital. The consecutive patients, who were scheduled to undergo a follow-up contrast-enhanced CT for the assessment of pain or the evaluation of suspected masses in organs, were prospectively enrolled in this study. In brief, nine variables were assigned as exclusion criteria: (1) a known history of hypersensitivity to iodine, (2) pregnancy, (3) lactation, (4) clinically unstable condition, (5) having received contrast media <7 days before the procedure, (6) cardiogenic shock, (7) known renal dysfunction, (8) pulmonary edema, (9) mechanical ventilator support. The subjects were randomized into two groups by the permuted block randomization method. Subjects allocated to the experimental arm underwent body computed tomography scanning using the contrast medium of iodixanol, whereas iohexol was used in the controlled arm of the trial.

### Methods

The radiology nurse established the heparin lock intravenous line before injection. Both of the contrast material were pre-warmed to 37 °C prior for intravascular use. The regional difference in contrast agent wash-in and wash-out time constants were identified as the main mechanism of delayed contrast enhancement on scan images. The highest attenuation difference for idoixanol on delayed contrast-enhanced images was achieved 4 min post injection comparing to 3 min for iohexol. In this trial, 1000 individuals were injected 100 ml iohexol 300 mg I/ml, while the other 1000 participants received intravenously 100 ml iodixanol 300 mg I/ml. The whole participants were monitored through completion of injections and throughout the scanning. In addition, these patients were further interviewed at the end of the test immediately, at 1 h, as well as at 24 h, concerning any local or systemic syndrome. The radiology nurse recorded vital signs at the time of interview. Urine and blood specimens were also obtained at the aforementioned time. The CTDIvol was 13.82 mGy for the 80 kV scan protocol.

### Laboratory analysis

This analysis was performed on both venous blood samples and voided urine samples, which were collected in a blinded fashion and measured by auto-analyzer of the clinical laboratory in Chongqing City Chinese Medicine Hospital. Generally, blood samples included tests for potassium chloride, platelet count, alanine aminotransferase, white blood cells, eosinophils, lymphocytes, aspartate aminotransferase, hemoglobin, red blood cells, sodium, blood urea nitrogen, creatinine, basophils, hematocrit, lactate dehydrogenase, and neutrophils. Urine samples usually contain occult blood (red blood cells), glucose, pH, specific gravity, and protein.

### Assessment of contrast enhancement on images

All participants were instructed to practice breath holding before scanning which was all performed on a second-generation 64-slice scanner. Three experienced CT radiologists, each with more than 5 years’ work experience was allocated in a group, to evaluate the images independently. Contrast quality grades should arrive at consensus for each diagnostic item by discussion. None radiologists had been involved in the patients recruiting. The radiologists were fully unaware of the original examination interpretation results and the specific contrast material injected. Contrast quality grades which is referenced as diagnostic images quality in this passage, which could be classified into three distinct grades as follows: Grade one is optimal, the one that provides optimal information to make a definitive decision. Grade two is the suboptimal choice, which provides less definitive information to make a suboptimal decision, which was also taken, if the images enhancement could not arrive at the optimal conditions in any aspect. Grade three is definitely not determinative, could not offer sufficient information to make the diagnosis. Results of this trial would be presented for all participants recruited in this research.

### Contrast agent reactions

Based on the reaction severity according to the criteria, patients’ reactions to the contrast enhanced CT could be graded as mild, moderate, or severe (Pintassilgo Santos et al. 2009). Discomfort related to the injection was classified into three grades as follows: mild, moderate, or severe, with the definition of subjective feeling, which resulted from physiology and psychology. Mild contrast reactions including rash, emesis, peculiar taste, itching, did not require any treatment. Moderate events were of any aforementioned in more advanced degree and required close monitoring and immediate therapy. Serious reactions were regarded as life-threatening events and more robust solutions were further required. All incidents accompanied with the material injection were recorded in this institution. For each adverse event, both supervising CT radiologists and technologists involved, completed the incidents reports, which contain the personal characteristics, contrast medium as well as date of the scanning and the events descriptions. These reactions could be classified into either immediate or delayed event according to the events onset time. Immediate events were those that occur within 1 h and were reported by experimentalists nearby or patients. Delayed reactions were subsequent clinical symptoms up to 7 days from time of intravenous use. The patients or their companions reported these information to us voluntarily.

### Statistical analysis

Quantitative variables were summarized by use of descriptive statistics, expressed as mean ± SD, while categorical variables were expressed as frequencies or percentages. Personal characteristics were compared between two contrast media by Student’s t test. Fisher’s exact test was used to evaluate the frequency of adverse events and discomfort. P values <0.05 were considered statistically significant difference. Statistical analysis was performed by spss version 18.0 for Mac.

### Ethics statement

This study was approved by local institutional review boards, and written informed consents were obtained from all patients before they were randomly assigned.

## Results

### Patients’ characteristics

**Fig. 1:**
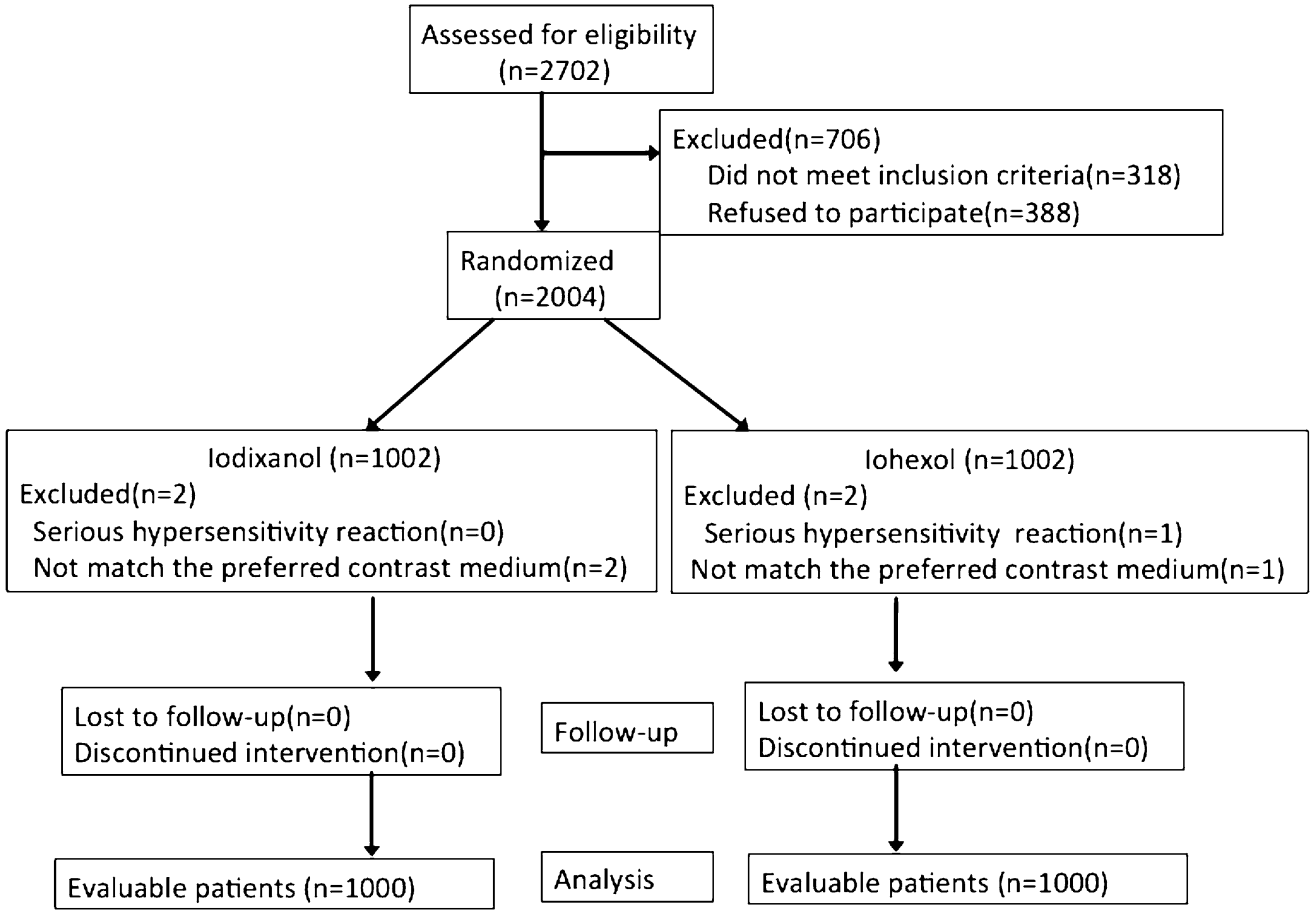
Flow of patients through the study

2702 participants were assessed for eligibility between April 2014 and 2015. Among these, 318 individuals met at least one of the specified exclusion criteria in this study, while 388 patients declined to participate the research. The other participants (n = 2004) were randomly assigned either to an iohexol or iodixanol program (Fig. 1). One were excluded, among these enrolled participants, on account of a serious hypersensitivity event in iohexol group and three were excluded because randomization assignment was not match the preferred contrast medium (one in iohexol and two in iodixanol group) of their own. No significant difference was detected between two groups for the demographic characteristics, as shown in Table 1.

**Table 1:** Patient demographic information

Safety parameter	Iodixol	Iohexol	P value
	n = 1000	n = 1000	
Male, n (%)	549 (54.9)	631 (63.1)	0.782
Age (years)	52 ± 13.3	55 ± 13.6	0.652
BMI (kg/m^2^)	25.2 ± 3.1	24.9 ± 2.8	0.263
Baseline SCr (mg/dl)	1.46 ± 1.2	1.53 ± 0.72	0.397
Baseline CrCl (ml/min)	47.51 ± 4.3	49.21 ± 5.7	0.421

### Image enhancement and quality

With regard to the overall enhancement, the diagnostic images were performed in CT room, which deployed the same monitors and similar machines. These images were diagnosed and classified by onsite-experienced investigators into three grades. As Table 2 shown, only 11 participate were regarded as Grade 3 in iodixanol group while 21 patients were considered as Grade 3 in iohexol group. On the contrary, the diagnostic images in iodixanol group was evaluated as Grade 1 for 80.9 % (809/1000) of the individuals and 82.6 % (826/1000) of the patients in the iohexol group. In Grade 2163 images were considered suboptimal in iodixanol group and 170 in iohexol. No significant difference was detected in image quality between contrast medium among the grades (P > 0.05, Table 1).

**Table 2:** Diagnostic image quality

	Iodixanol	Iohexol	P value
	n = 1000	n = 1000	
Grade 1	826 (82.6 %)	809 (80.9 %)	
Grade 2	163 (16.3 %)	170 (17 %)	0.178
Grade 3	11 (1.1 %)	21 (2.1 %)	

Comparison of the time duration with vascular attenuation values equal to or above 300 HU revealed no statistically significantly difference between iohexol and iodixanol for aorta (350 vs 293; P = 0.03, Table 3).

**Table 3:** CT HU for aorta

	Iodixanol	Iohexol	P value
HU	350 ± 25.7	293 ± 18.6	0.032

### Laboratory results

In brief, we did not detect any significant difference between the two groups in all laboratory results measured. Neither clinically significant changes nor any relevant trends, which demonstrated toxicity attributable to any contrast agents, were founded in these specific laboratory results (data not shown). However, among these patients, thirty patients in iohexol group had deviated from the maximum limits of normal reference range, and turned into normal in 3 days post-injection. The increase in serum creatinine was considered to be clinically relevant by radiologists. Ten patients in iodixanol group had increase in serum creatinine and decreased to normal reference range in 3 days post-injection. However, one participant receiving iodixanol got 1.3 mg/dl in creatinine level on the day prior to the research, and 1.8 mg/dl after 4 days. He received low dose gentamycin, and ampicillin therapy with the decrease of creatinine 2 days later. Summary results for creatinine are presented in Table 4.

**Table 4:** Summary values for patient creatinine levels

Serum creatinine (mg/dl)	Iodixanol group	Iohexol group	P value
Baseline level	1.46 ± 1.23	1.53 ± 0.72	0.397
At day 2	1.43 ± 1.52	1.56 ± 0.68	0.432
At day 3	1.43 ± 1.61	1.58 ± 0.59	0.451
>10 % rise in creatinine	14	27	0.028
>25 % rise in creatinine	1	1	1

### Safety results

The overall incidence of adverse reaction was low for iodixanol (0.9 %). A nearly threefold increase in the overall adverse events frequency was detected with iohexol (2.5 %), which was significant (P < 0.05, Fisher’s exact test). Both of the immediate adverse events and delayed ones experienced increased in iohexol groups compared with iodixanol, while the changes were not significant (P > 0.05). Though immediate adverse reactions predominated in both contrast materials, the rate of delayed adverse reactions was much greater with iohexol group (P > 0.05). For both mediums, most of the adverse reactions were of mild severity (87 % iohexol vs 89 % iodixanol). For moderate events combined, the percentage of iodixanol was higher than iohexol (2.4 % iohexol vs 1 % iodixanol). Futher, only one patients suffered severe side effects after injecting iohexol. For both agents, neither a monthly nor a seasonal trend in the incidence of adverse reactions during the study period was included in the study. The results of this study are summarized in Table 5.

**Table 5:** Patients safety results

Safety parameter	Iodixanol	Iohexol	P value
Total			
Adverse events	9	25	0.04
Immediate events	7	20	0.018
Delayed events	2	5	0.452
Severity			
Mild	8	22	NA
Moderate	1	2	NA
Severe	0	1	NA

The profile of adverse reactions is similar in two groups. Almost all adverse reactions had a cutaneous component (75 % in iohexol vs 90 % in iodixanol, data not shown), with most commonly urticaria. However, respiratory and gastrointestinal was the next most frequent symptom, and the frequency of them was higher in iohexol group. In addition, two severe reaction was observed with iohexol while none was found with iodixanol. In this particular event, both of the patients developed erythemas immediately, have profound hypotension, loss of consciousness at the injection of iohexol. Luckily, they responded to administration of oxygen, fluid resuscitation, and epinephrine. After transferring to the emergency department, the patients made a complete recovery. No deaths or other severe events occurred in this study. Pulse rate as well as systolic and diastolic blood pressure showed transient, mild variation within normal ranges for almost all individuals in all recordings. Mean values of all procedures stayed ordinarily within a single standard deviation of means. The five patients who showed greater ranges were diagnosed to be clinically non-relevant by two cardiologists. No significant of vital signs and the relation between types of contrast material injected could be detected.

In the follow-up research, one patient had severe skin rash 2 days after injecting iohexol. The skin lesion had pruritus, edema, erythema, urticaria, and exanthema. Receiving low dose (0.5 mg/kg) oral prednisolone therapy result in the resolution of skin lesion 1 week later. All researches were successfully completed in all of the enrolled individuals.

## Discussion

The data of this research detected similar diagnostic quality enhancement by iohexol and iodixanol. The comparison was performed by paralleled total iodine amounts and identical iodine delivery rates under standardized conditions in intra-individuals. The overall diagnostic quality was evaluated as optimal in 80.9 % (809/1000) of the individuals receiving iodixanol and 82.6 % (826/1000) of the groups injecting iohexol. We did not detect any significant difference in diagnostic image enhancement among the three grades between contrast medium (P < 0.05). In addition, The impact of iodixanol also persisted longer than the other agent. The result is similar to the detection of Elmstahl et al. (2007). The detection might also indicate that the increased quality enhancement observed with iodixanol injection might provide improved images diagnosis and detailed descriptions of several organic disease. More importantly, the iso-osmotic agent might be more efficient and the smaller doses of iodixanol could pose similar diagnostic enhancement compared with larger doses of the other one when imaging some specific organ.

There are distinct types of complications following intravenous use: soft tissue injury, direct chemical toxicity, nephrotoxicity, and anaphylactic reactions (Leow et al. 2015; Li et al. 2015; McAlister and Kissane 1990). Nearly 2 % of the patients in this study experience adverse reactions related with intravenous contrast medium, similar to the dictation of Ho et al (Ho et al. 2007). Late reactions are reported with frequencies nearly 20 %. Our observation indicates that compared to iohexol, iodixanol is associated with a lower frequency of late reaction (P < 0.05). These results suggested that there exists the increased frequency of late reaction with hypotonic contrast medium compared to hyperosmotic contrast medium. This finding is also consistent with the literature that suggests that delayed reactions are more common with nonionic dimers than with monomers (Webb et al. 2003). Definitely, the physiological background of delayed reaction is largely unknown. In this study, the incidence of allergic reaction was 2 % among all paticipants, slightly less than previous study (Li et al. 2015). In all, 0.2 % patients with iodixanol and 0.5 % with iohexol had late allergic reaction. These results is similar to the report that the adverse reaction was minimal for patients receiving iodixanol compared with individuals injecting iohexol (Chalmers and Jackson 1999). During this study, no significant differences were account for the different frequency of adverse reactions in the methods of reporting adverse events with either contrast medium.

Contrast nephropathy (CN) is the complex complications of arteriography action, which could be defined as the acute renal function impairment, caused by contrast material injection (McCullough et al. 1997; Lindholt 2003). The clinical presentation of CN is distinct, having a temporal relation with the high-risk patients groups and the onset of a serum creatinine levels increase within the 24 h to 5 days following the injection. CN could be diagnosed if the value of serum creatinine is rising by 0.5 mg/dl or greater than 25 % of baseline or more. A previous study has demonstrated that iohexol was slightly more nephrotoxic compared with iodixanol (Chalmers and Jackson 1999). Accordingly, in this research, 10 % patients receiving iodixanol had an increase in serum creatinine greater than 10 % of baseline in the week following scanning, compared with 15 % in the iohexol group (P > 0.05). Several previous studies have demonstrated that contrast medium could pose direct cytotoxic effects on renal structures, such as glomerulus, kidney tubules. Until now, several hypothesis have been proposed to explore the further mechanisms, including the discovery of disturbed renal perfusion/hypoxia, the renal tubular epithelium caused by direct toxicity of contrast medium, or apoptosis of glomerular, and altered glomerular function (Gyoten 1998; Hizoh and Haller 2002; Duarte et al. 1999; Tervahartiala et al. 1993). However, there is no evidence that the use of any medicine could provide any significant prophylactic benefit, based on these mechanisms. The serious complications in the high-risk patients’ groups, including anaphylactic shock, acute renal failure, still should be managed by hemodialysis. Thus, further measures and researches about cytokines and complement profiles are needed in sequential studies.

A limitation of our research, consistent with previous contrast material clinical trials, is the comparatively small patients population with special species of chronic disease, such as diabetes and hypertension without CKD. The previous studies has indicated that non-diabetic patients with preexisting renal insufficiency and patients with diabetes mellitus are conditions clinically carrying a high risk of nephrotoxicity events resulted by contrast material (Gavant and Siegle 1992). In this research, the data of body CT scanning revealed that iodixanol is superior to iohexol in safe and efficacy in the concentrations tested with 15 diabetes mellitus and 10 renal insufficiencies. In the iodixanol group, none diabetic patient with renal insufficiency had CN while five diabetes mellitus with normal renal function experience CN in the iohexol group. However, there were no significant difference in serum creatinine levels, and no death adverse reactions occurred in these overall samples. Our experience is mirrored by that of others who have used iodixanol for excretory urography (Gavant and Siegle 1992). Although these special patient populations were small, and the broad safety conclusions of these two contrast agents are beyond the scope or intent of this study, we do suppose that further researches are needed to add to the validity of this study in terms of applicability to clinical practice.

Above all, there were no apparent differences in the diagnostic images enhancement. The incidence of adverse reactions with the universal use of iodixanol for CT were lower compared with iohexol and within the range of previously reported incidences for other contrast material. In addition, in our experience, the rate of immediate and delayed adverse reactions were significantly greater with iohexol than iodixanol. No seasonal influence was detected to affect the incidence of adverse events compared with either contrast material. Additional studies will be needed to further illuminate the iodixanol clinical properties.
